# Supplementary material for: Marine Bacteria from Rocas Atoll as a Rich Source of Pharmacologically Active Compounds
Source: Mar Drugs. 2019 Nov 28;17(12):671. doi: 10.3390/md17120671 (PMC6949966; doi:10.3390/md17120671)

SUPPLEMENTARY MATERIAL

Marine Bacteria from Rocas Atoll as a rich source of pharmacologically active compounds

Karen Y. Velasco-Alzate[1#], Anelize Bauermeister[1,2#], Marcelo M. P. Tangerina[3], Tito M. C. Lotufo[4], Marcelo J. P. Ferreira[3], Paula C. Jimenez[5], Gabriel Padilla[6], Norberto P. Lopes[2], Letícia V. Costa-Lotufo[1*]

[1] Departamento de Farmacologia, Instituto de Ciências Biomédicas, Universidade de São Paulo, São Paulo/SP, Brazil; karenvelasco20@gmail.com (K.Y.V.A.); ane.meister@usp.br (A.B.); costalotufo@gmail.com (L.V.C.L.)

[2] NPPNS, Departamento de Física e Química, Faculdade de Ciências Farmacêuticas de Ribeirão Preto, Universidade de São Paulo, Ribeirão Preto/SP, Brazil; npelopes@fcfrp.usp.br (N.P.L.)

[3] Departamento do Botânica, Instituto de Biociências, Universidade de São Paulo, São Paulo/SP, Brazil; marcelotangerina@usp.br (M.M.P.T.); marcelopena@ib.usp.br (M.J.P.F.)

[4] Departamento de Oceanografia Biológica, Instituto Oceanográfico, Universidade de São Paulo, São Paulo/SP, Brazil; tmlotufo@gmail.com (T.M.C.L.)

[5] Departamento de Ciências do Mar, Universidade Federal de São Paulo, Santos/SP, Brazil; paulacjimenez@gmail.com (P.C.J.)

[6] Departamento de Microbiologia, Instituto de Ciências Biomédicas, Universidade de São Paulo, São Paulo/SP, Brazil; gpadilla@icb.usp.br (G.P.)

***** Correspondence: costalotufo@gmail.com; Tel.: +55-11-30917316

#authors with equal contribution

Table S1. Bacteria strains isolated from ascidians, sponges and sediments from the Rocas Atoll and their anticancer activity results against human colon carcinoma cell line HCT-116. Extracts were considered cytotoxic when inhibited over 75% of the cell growth at 50 μg/mL.

Strain	Sample Source	Host Species	Bacteria Identification[1]	Sequence Accession number[2]	Cell Growth Inhibition at 5 μg/mL (%)[3]	Cell Growth Inhibition at 50 μg/mL (%)[3]
**BRB-256**	Ascidian	*Euherdmania* sp.	*Salinispora* sp.	MK720164	47.40	94.89
**BRB-283**	Ascidian	*Didemnum granulatum*	-	-	25.30	64.82
**BRB-284**	Ascidian	*Euherdmania* sp.	*Salinispora* sp.	MK720165	64.43	91.43
**BRB-285**	Ascidian	Sp. nov 3	-	-	67.9	70.00
**BRB-288**	Ascidian	*Trididemnum rocasensis*	-	-	48.25	56.56
**BRB-297**	Ascidian	*Trididemnum rocasensis*	-	-	0.00	16.56
**BRB-298**	Ascidian	Sp. nov 3	*Streptomyces* sp.	MK720171	99.34	99.97
**BRB-301**	Ascidian	*Polysyncraton maurizeliae*	-	-	24.51	40.19
**BRB-302**	Ascidian	Sp. nov 1	*Streptomyces* sp.	MK720166	100.00	100.00
**BRB-304**	Ascidian	Sp. nov 3	-	-	0.00	67.43
**BRB-306**	Ascidian	*Polysyncraton maurizeliae*	-	-	0.00	60.76
**BRB-307**	Ascidian	*Euherdmania* sp.	-	-	8.19	52.08
**BRB-308**	Ascidian	*Ascidia viridina*	-	-	0.85	50.38
**BRB-309**	Ascidian	*Didemnum granulatum*	-	-	6.81	40.57
**BRB-311**	Ascidian	*Euherdmania* sp.	-	-	15.54	44.79
**BRB-314**	Ascidian	Sp. nov 2	-	-	15.75	55.10
**BRB-320**	Ascidian	*Euherdmania* sp.	*Salinispora* sp.	MK720162	69.12	96.37
**BRB-324**	Ascidian	Ascidia viridina	-	-	18.44	50.69
**BRB-326**	Ascidian	Sp.nov 3	-	-	0.00	0.00
**BRB-346**	Sediment	-	-	-	21.00	30.00
**BRB-349**	Sponge	*Plakinastrella microspiculifera*	*Nocardiopsis* sp.	MK720152	24.00	76.09
**BRB-350**	Sponge	*Plakinastrella microspiculifera*	*Streptomyces* sp.	MK720170	81.03	97.24
**BRB-351**	Sediment	-	-	-	22.16	69.92
**BRB-352**	Sediment	-	*Nocardiopsis* sp.	MK720153	77.74	77.39
**BRB-353**	Sponge	*Plakinastrella microspiculifera*	-	-	44.71	51.68
**BRB-354**	Sediment	-	-	-	23.34	71.20

**BRB-355**	Sponge	*Plakinastrella microspiculifera*	*Nocardiopsis* sp.	MK720167	30.80	77.98
**BRB-356**	Sponge	*Plakinastrella microspiculifera*	*Streptomyces* sp.	MK720154	79.47	91.72
**BRB-357**	Sponge	*Chondrilla cf. nucula*	-	-	20.86	73.69
**BRB-358**	Sponge	*Plakinastrella microspiculifera*	*Streptomyces* sp.	MK720163	74.35	83.62
**BRB-359**	Sediment	-	-	-	27.63	56.85
**BRB-360**	Sediment	-	-	-	18.97	35.74
**BRB-361**	Sponge	*Chondrilla cf. nucula*	-	-	16.38	49.59
**BRB-362**	Sponge	*Chondrilla cf. nucula*	-	-	0.00	0.85
**BRB-363**	Sponge	*Chondrilla cf. nucula*	-	-	28.38	59.91
**BRB-364**	Sponge	*Chondrilla cf. nucula*	*Nocardiopsis* sp.	MK720156	26.23	88.21
**BRB-366**	Sponge	*Chondrilla cf. nucula*	-	-	62.05	62.05
**BRB-367**	Sponge	*Chondrilla cf. nucula*	-	-	22.63	40.97
**BRB-368**	Sponge	*Chondrilla cf. nucula*	*Brevibacterium* sp.	MK720174	n.d	90.85
**BRB-371**	Sponge	*Chondrilla cf. nucula*	-	-	33.54	36.76
**BRB-372**	Sponge	*Chondrilla cf. nucula*	-	-	11.16	14.34
**BRB-373**	Ascidian	*Tridemnum Maragogi*	-	-	19.92	43.91
**BRB-374**	Sponge	*Chondrilla cf. nucula*	-	-	22	49
**BRB-375**	Sponge	*Plakortis* sp.	-	-	22.10	51.15
**BRB-383**	Sponge	*Plakinastrella microspiculifera*	-	-	2.96	68.93
**BRB-385**	Sponge	*Chondrilla cf. nucula*	-	-	23.43	56.04
**BRB-386**	Sponge	*Plakinastrella microspiculifera*	-	-	51.21	65.49
**BRB-388**	Sediment	-	-	-	16.06	41.99
**BRB-391**	Ascidian	*Tridemnum Maragogi*	*Bacillus* sp.	MK720168	85.11	100.00
**BRB-392**	Sediment	-	-	-	4.55	49.71
**BRB-393**	Sediment	-	-	-	0.00	6.31
**BRB-397**	Sponge	*Chondrilla cf. nucula*	*Bacillus* sp.	MK720169	35.89	92.40
**BRB-398**	Sediment	-	-	-	2.08	16.50
**BRB-399**	Sponge	*Chondrilla cf. nucula*	*Salinispora* sp.	MK720158	69.93	82.96
**BRB-405**	Ascidian	*Tridemnum Maragogi*	-	-	14.36	23.00
**BRB-406**	Ascidian	*Tridemnum Maragogi*	-	-	73.28	94.78
**BRB-407**	Ascidian	*Tridemnum Maragogi*	-	-	80.94	92.93

**BRB-408**	Sediment	-	*Bacillus* sp.	MK720155	88.94	100.97
**BRB-412**	Ascidian	*Trididemnum Maragogi*	-	-	9.45	46.35
**BRB-414**	Ascidian	*Trididemnum Maragogi*	*Salinispora* sp.	MK720161	86.52	98.86
**BRB-415**	Ascidian	*Trididemnum Maragogi*	*Salinispora* sp.	MK720172	64.51	97.97
**BRB-417**	Sediment	-	*Streptomyces* sp.	MK720159	17.21	95.73
**BRB-418**	Sediment	-	*Streptomyces* sp.	MK720157	34.44	98.88
**BRB-455**	Sediment	-	-	-	12.00	35.00
**BRB-456**	Sediment	-	-	-	21.04	71.98
**BRB-457**	Sediment	-	-	-	23.19	67.36
**BRB-458**	Sediment	-	-	-	14.63	27.64
**BRB-462**	Ascidian	*Trididemnum Maragogi*	*Salinispora* sp.	MK720160	75.39	92.81
**BRB-463**	Sponge	*Chondrilla cf. nucula*	-	-	29.00	57.00
**BRB-466**	Sponge	*Plakortis sp.*	-	-	21.42	49.35
**BRB-468**	Sponge	*Chondrilla cf. nucula*	*Salinispora* sp.	MK720173	61.19	94.04
**BRB-474**	Sponge	*Chondrilla cf. nucula*	-	-	14.67	25.07
**BRB-475**	Sediment	-	-	-	0.00	21.23
**BRB-476**	Sediment	-	-	-	14.28	20.82
**BRB-502**	Ascidian	*Trididemnum Maragogi*	-	-	16.77	29.52
**BRB-504**	Ascidian	*Trididemnum Maragogi*	-	-	44.10	102.30

[1]Bacterial identification based on 16S rRNA sequencing for those strains considered active

[2]Available at (http://www.ncbi.nlm.nih.gov/genbank).

[3]Cell growth inhibition was obtained by MTT assay, using HCT-116 colon cancer cells, after 72h incubation.

n.d. – not determined

Figure S1. Origin of the 80 bacterial strains recovered from the Atlantic Rocas Atoll. (A) Bacteria recovered from ascidians (40%), sponges (35%) and sediments (25%); (B) Identification of ascidians, sponge and sites where sediments were collected.

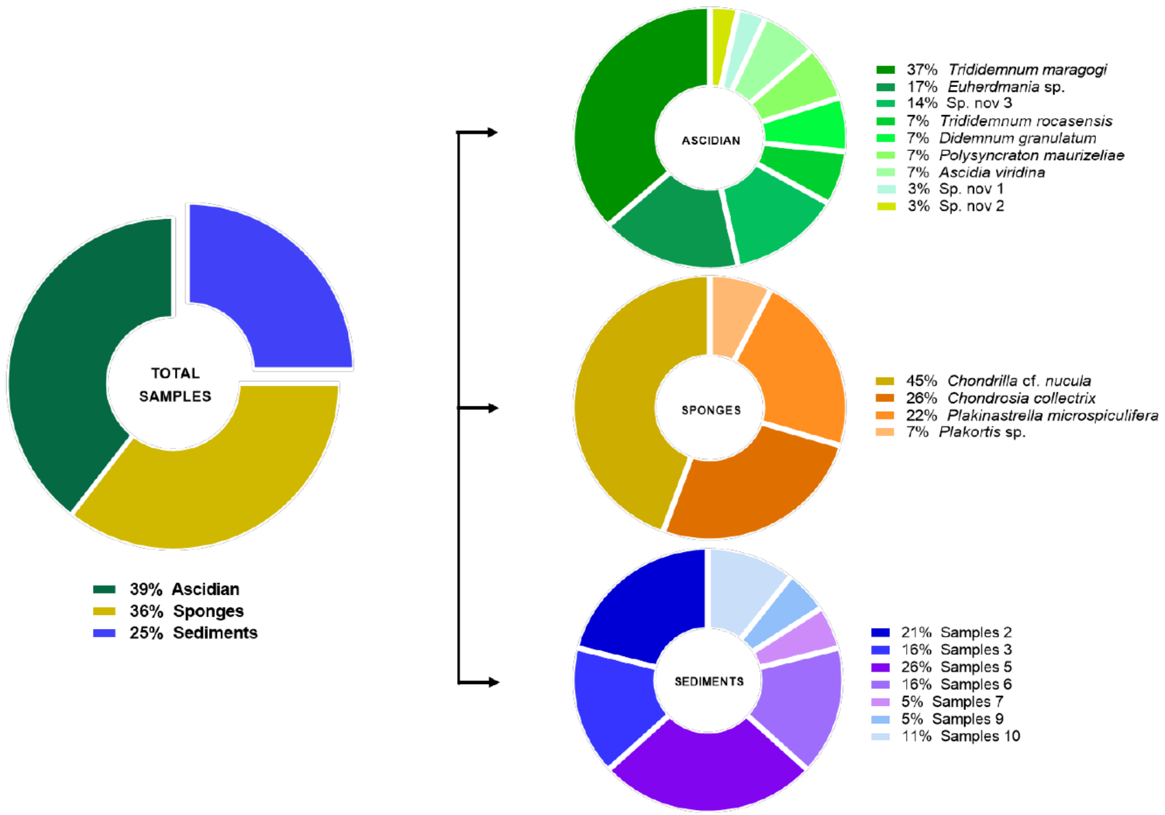

Figure S2. Molecular Network of crude extracts produced by bacteria recovered from Rocas Atol, using positive ionization mode (ESI^+) data. Node colors represent the strains extracts accordingly to the legend. Nodes highlighted in black boxes represent parent ions that are identified by GNPS library, and blue box represent cluster of ions that are present only in active crude extracts.

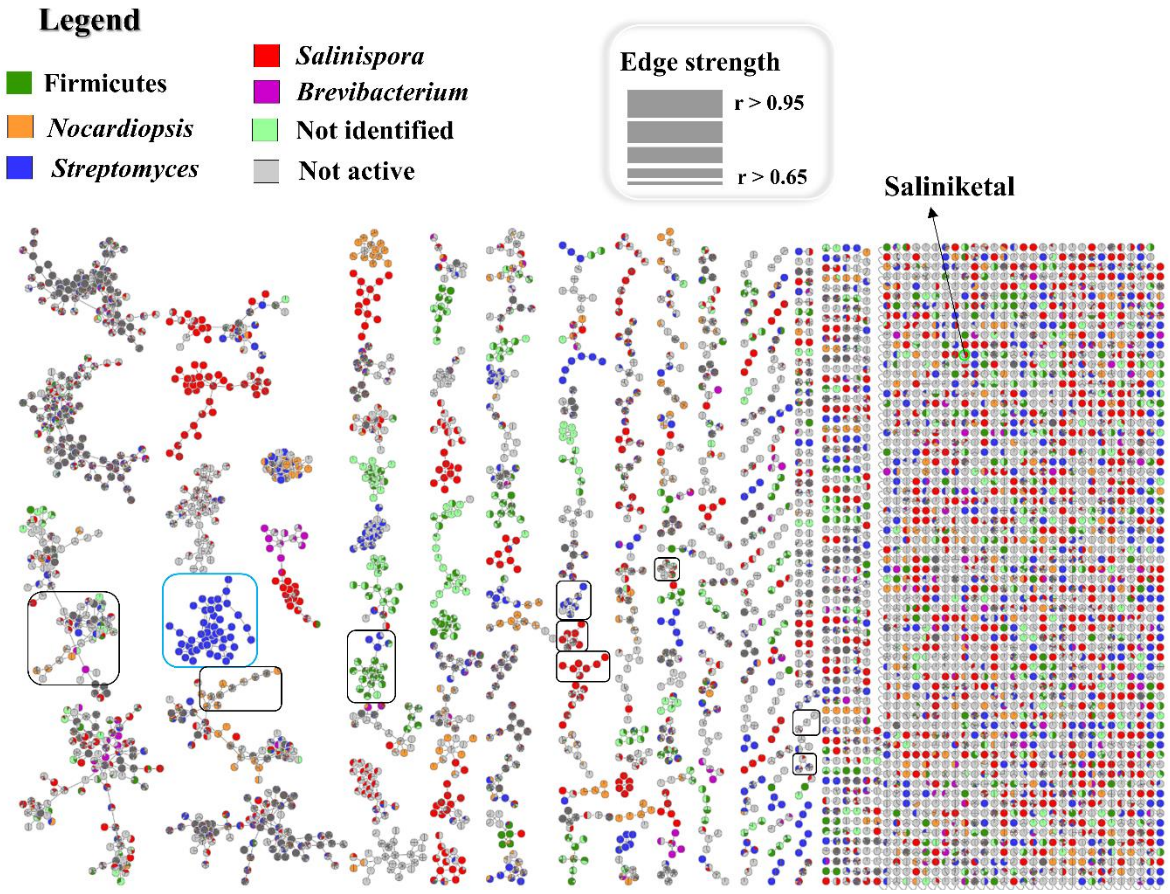

Figure S3. Cluster of the diketopiperazine family, observed as protonated adducts ([M+H]$^{+}$), produced by all investigated strain. The node color represents the genus of the bacteria, and edge size is according to the cosine score. The annotated nodes are circled in green.

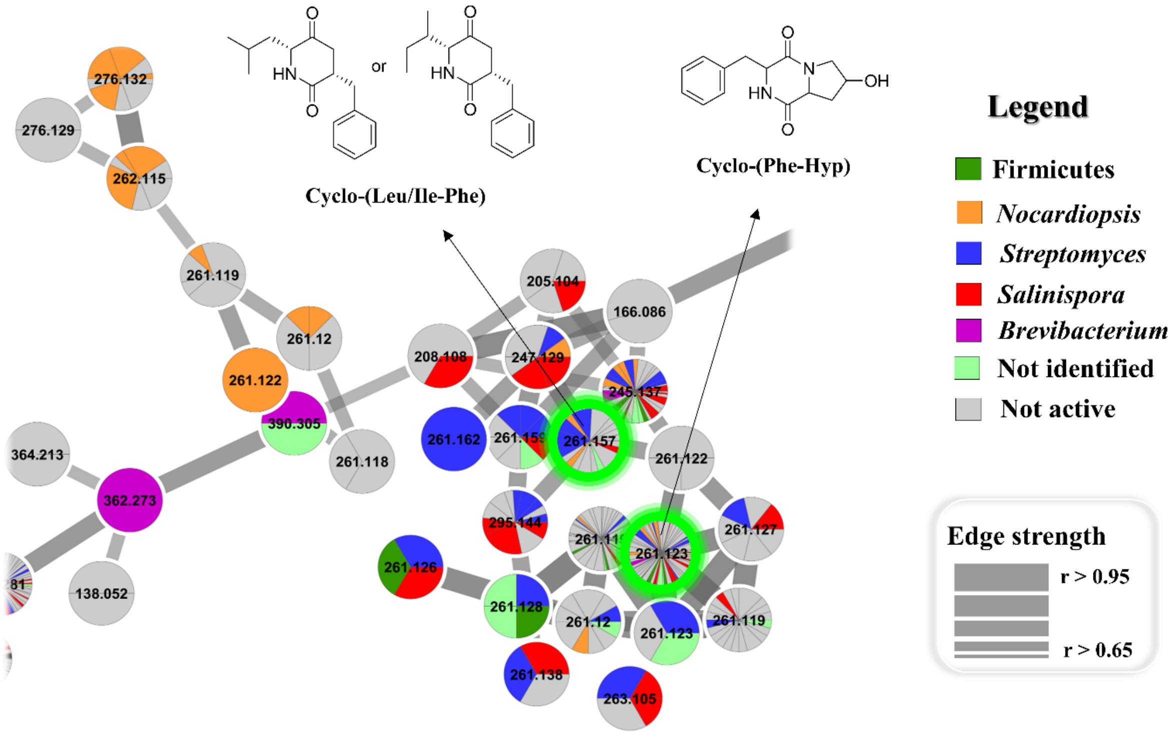

Figure S4. Cluster of the rifamycin family, observed as protonated adducts ([M+H]$^+$), produced mainly by *Salinispora* (BRB-256, BRB-399, BRB-414, BRB-415 and BRB-468), but also by BRB-283, a not active strain. The node color represents the genus of the bacteria, and edge size is according to the cosine score. The annotated node is circled in green.

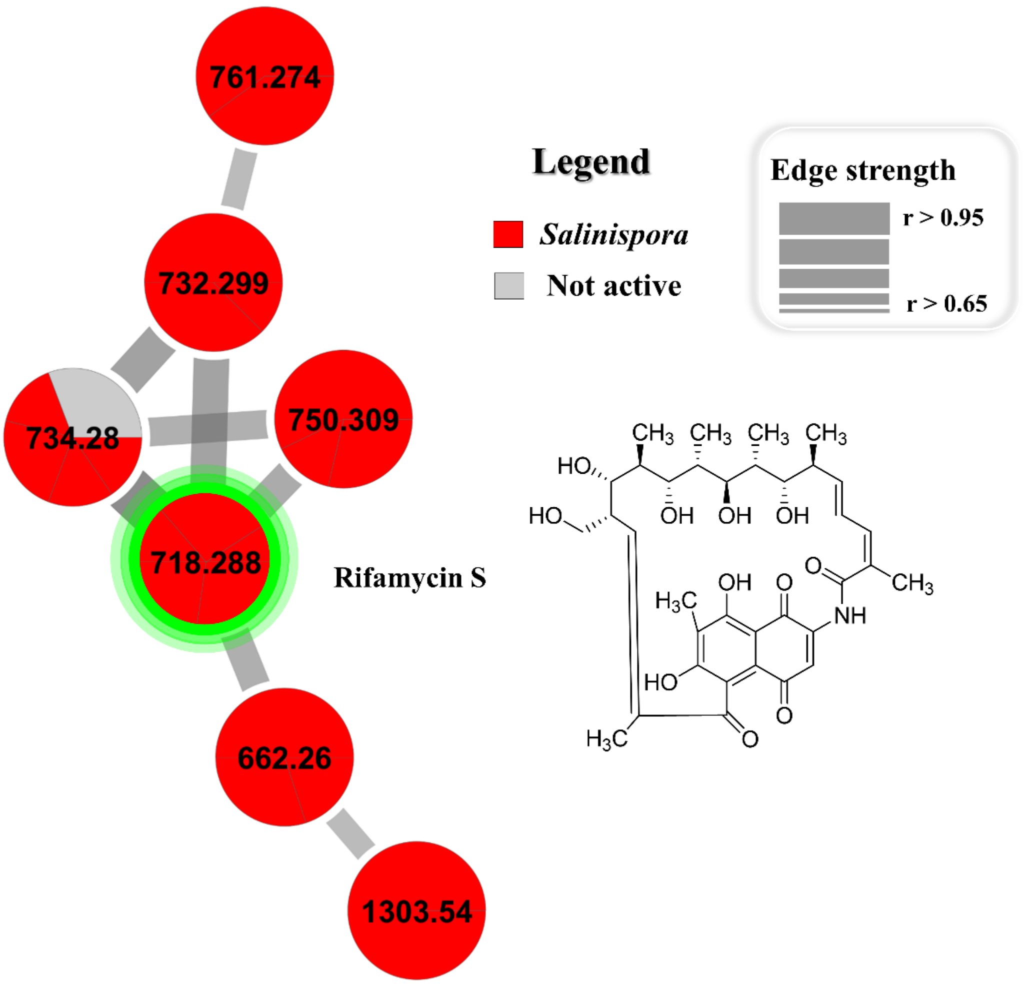

Figure S5. Cluster of lipopeptide family, including surfactins and esperin, observed as protonated adducts ([M+H]$^+$), produced by *Bacillus* (Firmicutes) (BRB-391, BRB-397 and BRB-408), *Nocardiopsis* (BRB-364), *Streptomyces* (BRB-298, BRB-302, BRB-350, BRB-356, BRB-358, BRB-417 and BRB-418), *Salinispora* (BRB-320 and BRB-415) not identified (BRB-406, BRB-407 and BRB-504) and not active (BRB-346 and BRB-359) strains. The node color represents the genus of the bacteria, and edge size is according to the cosine score. The annotated nodes are circled in pink.

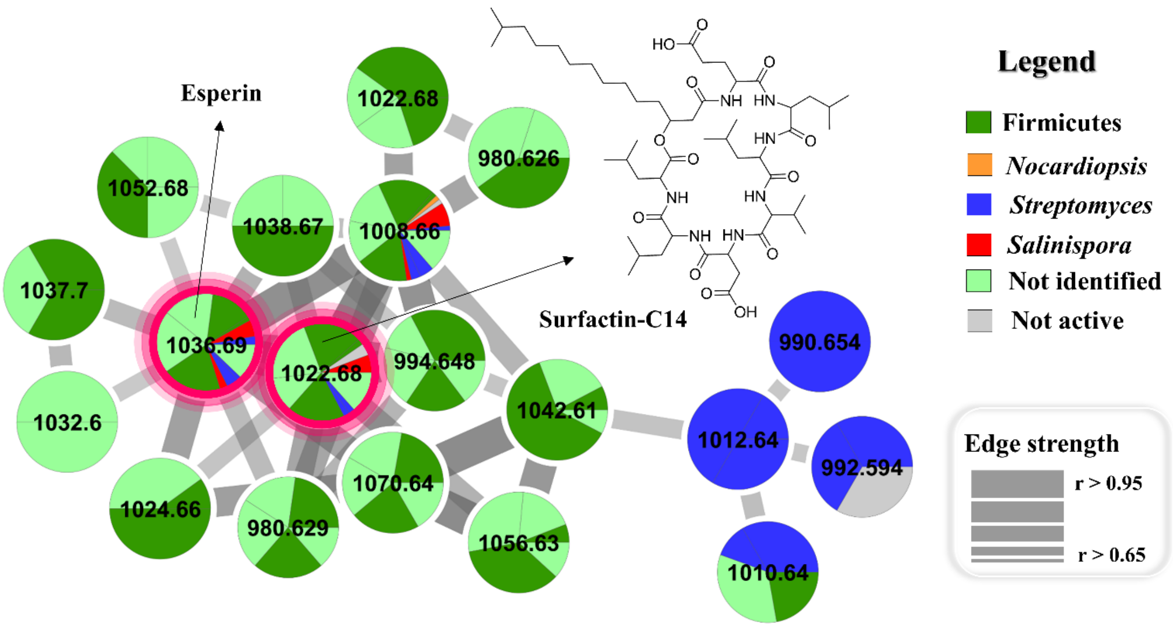

Figure S6. Cluster of the erythromycin family, observed as protonated adducts ($[M+H]^+$), produced mainly by not identified (BRB-346, BRB-374, BRB-375, BRB-405, BRB-455 and BRB-463) strains, but also by one *Salinispora* (BRA-415) strain. The node color represents the genus of the bacteria, and edge size is according to the cosine score. The annotated node is circled in green.

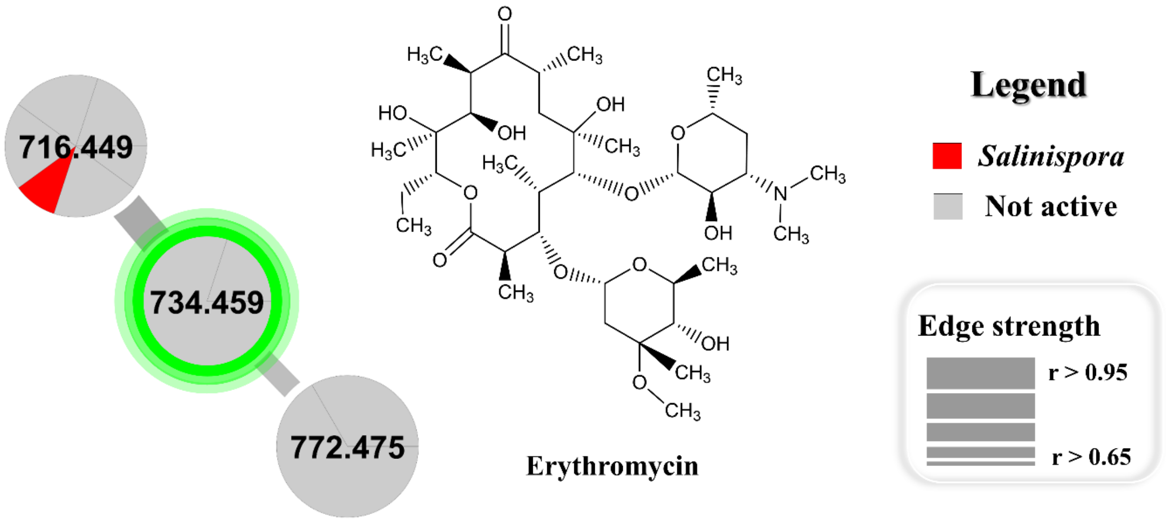

Figure S7. Cluster of the antibiotic TAN family, observed as protonated adducts ($[M+H]^+$), produced mainly by *Nocardiopsis* (BRB-349, BRB-352, BRB-355 and BRB-364), but also Firmicutes (BRB-397), *Streptomyces* (BRB-302 and BRB-358), *Salinispora* (BRB-414 and BRB-468), not identified (BRB-407) and not active (BRB-351, BRB-354, BRB-360, BRB-361, BRB-371, BRB-372, BRB-373, BRB-383, BRB-385, BRB-388, BRB-393 and BRB-455) strains. The node color represents the genus of the bacteria, and edge size is according to the cosine score. The annotated node is circled in green.

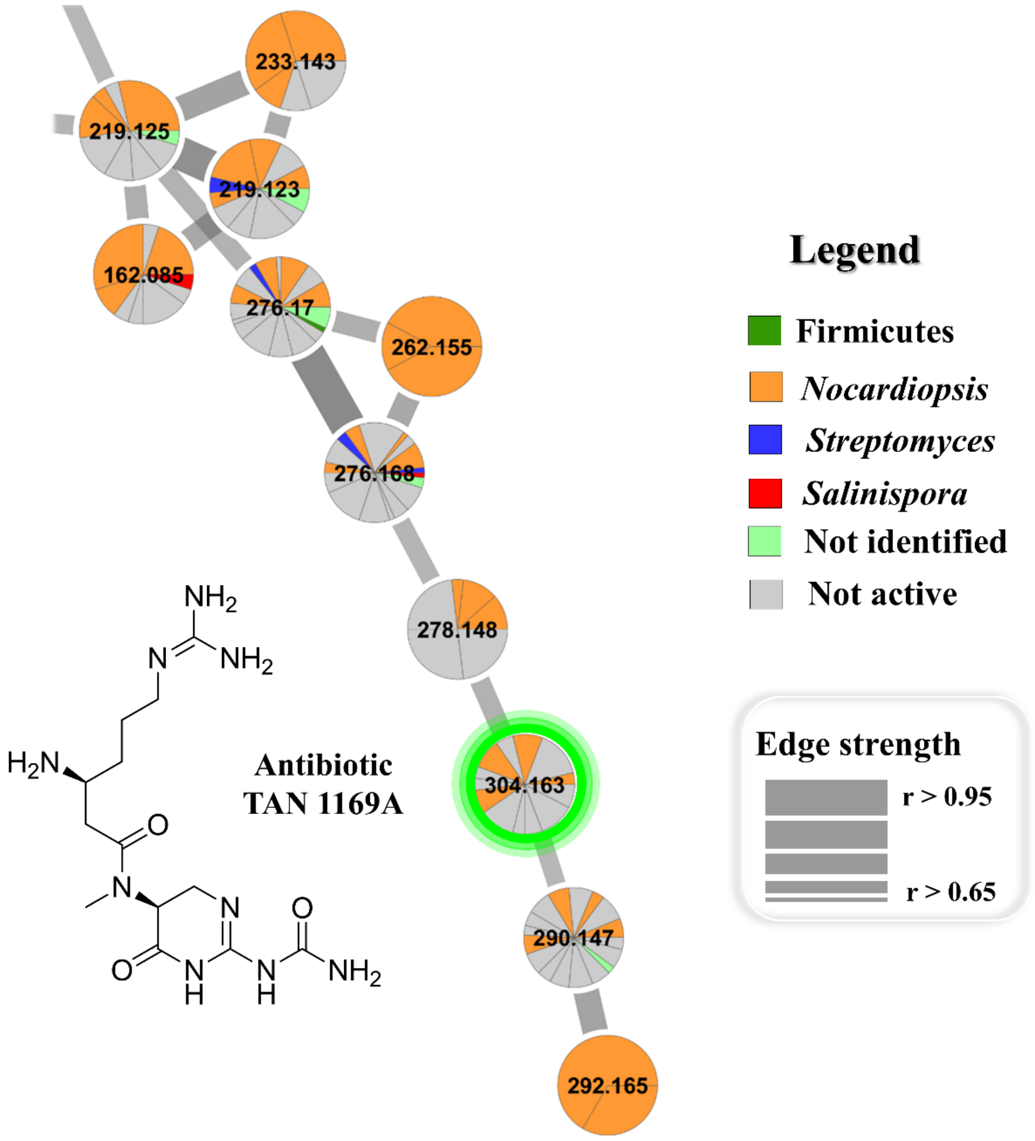

Figure S8. Cluster of the surugamide family, observed as protonated adducts ($[M+H]^+$), produced mainly by *Streptomyces* strains (BRB-350, BRB356 and BRB-358), *Nocardiopsis* (BRB-352) and not active (BRB-353, BRB-366, BRB-375 and BRB-386). The node color represents the genus of the bacteria, and edge size is according to the cosine score. The annotated nodes are circled in green.

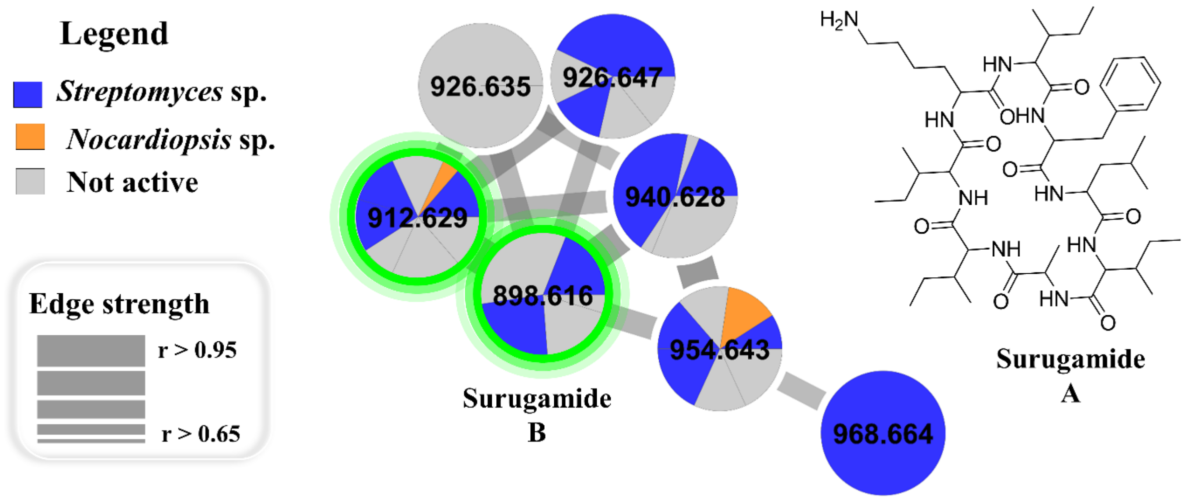

Figure S9. Cluster of the staurosporine family, observed as protonated adducts ($[M+H]^+$), produced mainly by *Salinispora* (BRB-256, BRB-320, BRB-399, BRB-414, BRB-415, BRB-462 and BRB-468), *Streptomyces* (BRB-302) not active (BRB-285, BRB-304, BRB-306, BRB-307, BRB-308, BRB-309, BRB-324 and BRB-405) strains. The node color represents the genus of the bacteria, and edge size is according to the cosine score. The annotated nodes are circled in green.

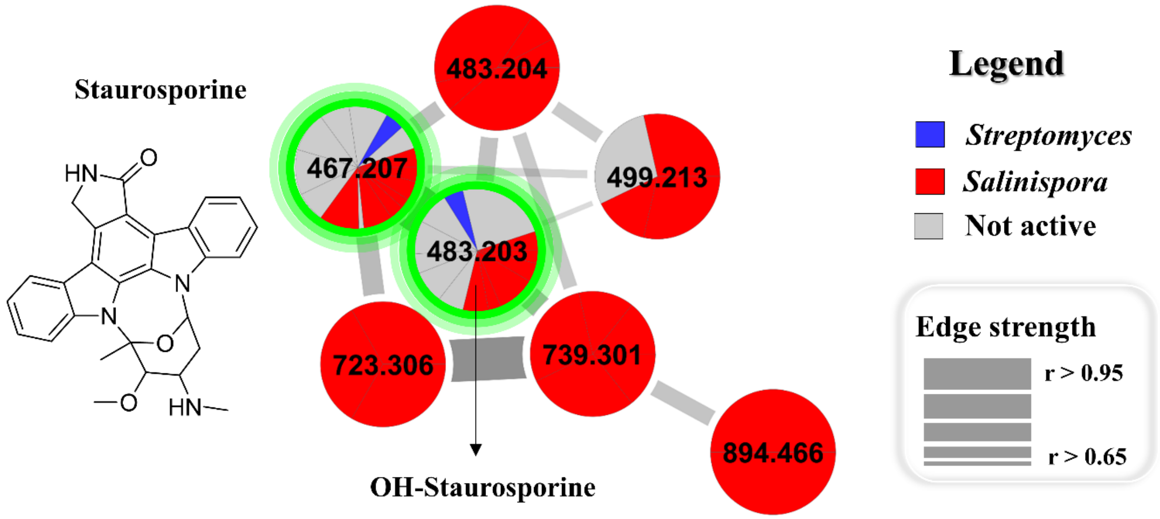

Figure S10. Cluster of the desferrioxamine family, observed as protonated adducts ($[M+H]^+$), produced mainly by not active (BRB-367, BRB-375, BRB-386, BRB-412, BRB-463 and BRB-476) strains, but also by *Streptomyces* (BRB-350 and BRB-358) and *Salinispora* (BRB-414). The node color represents the genus of the bacteria, and edge size is according to the cosine score. The annotated nodes are circled in green.

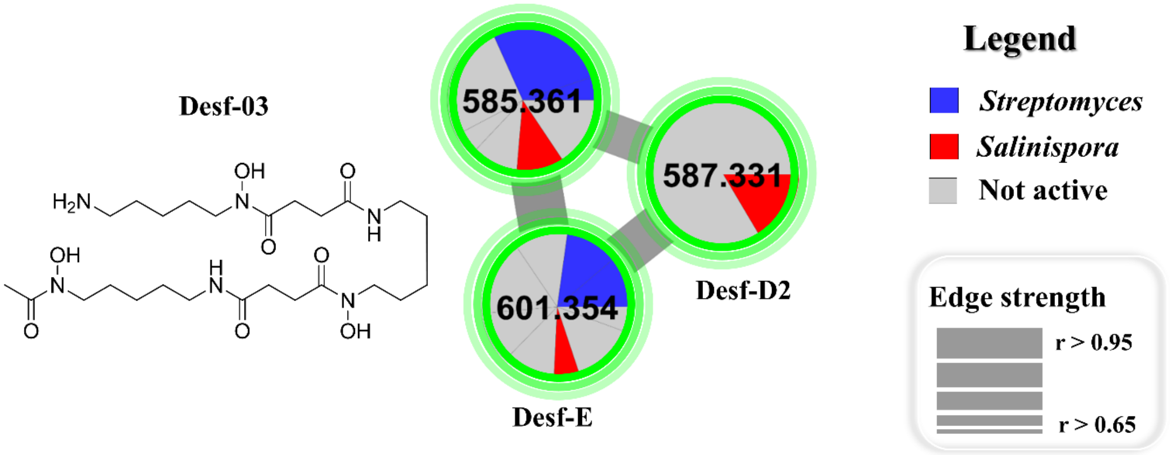

Figure S11. Cluster of the sphinganine family, observed as protonated adducts ($[M+H]^+$), produced by many different strains, including *Bacillus* (Firmicutes) (BRB-408), *Brevibacterium* (BRB-368), *Nocardiopsis* (BRB-364), *Salinispora* (BRB-256, BRB-414 and BRB-415), *Streptomyces* (BRB-298), not identified (BRB-406 and BRB-504) and not active (BRB-297, BRB-304, BRB-306, BRB-307, BRB-308, BRB-309, BRB-346, BRB-374, BRB-398, BRB-405 and BRB-463). The node color represents the genus of the bacteria, and edge size is according to the cosine score. The annotated node is circled in green.

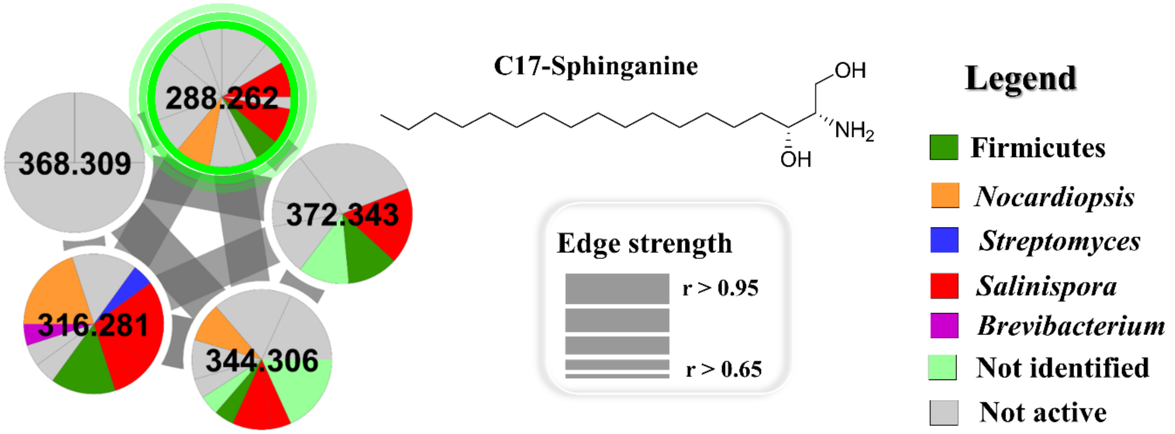

Figure S12. (A) Venn diagram and (B) distributions of samples according to the number of ions detected in cytotoxic and non-cytotoxic extracts; (C) Shannon-Wiener index. For these analyses, it was used the .csv table obtained from the molecular network containing the features information.

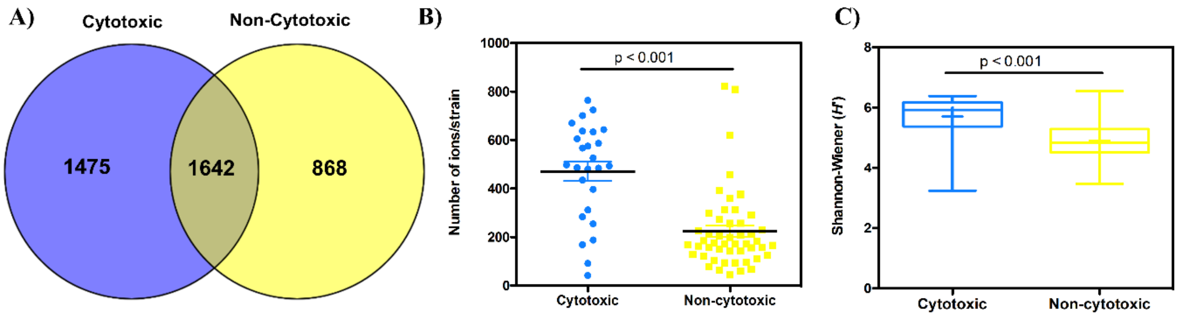

Figure S13. (a) Chromatographic profile of the extract produced by Streptomyces BRB-302 in A1 culture medium. Dashed line set the fractions collected over time. (b) Growth inhibition response in cytotoxic assay using the human colon adenocarcinoma HCT-116 cell line. In red are highlighted the fractions that contain the novonestmycin derivatives.

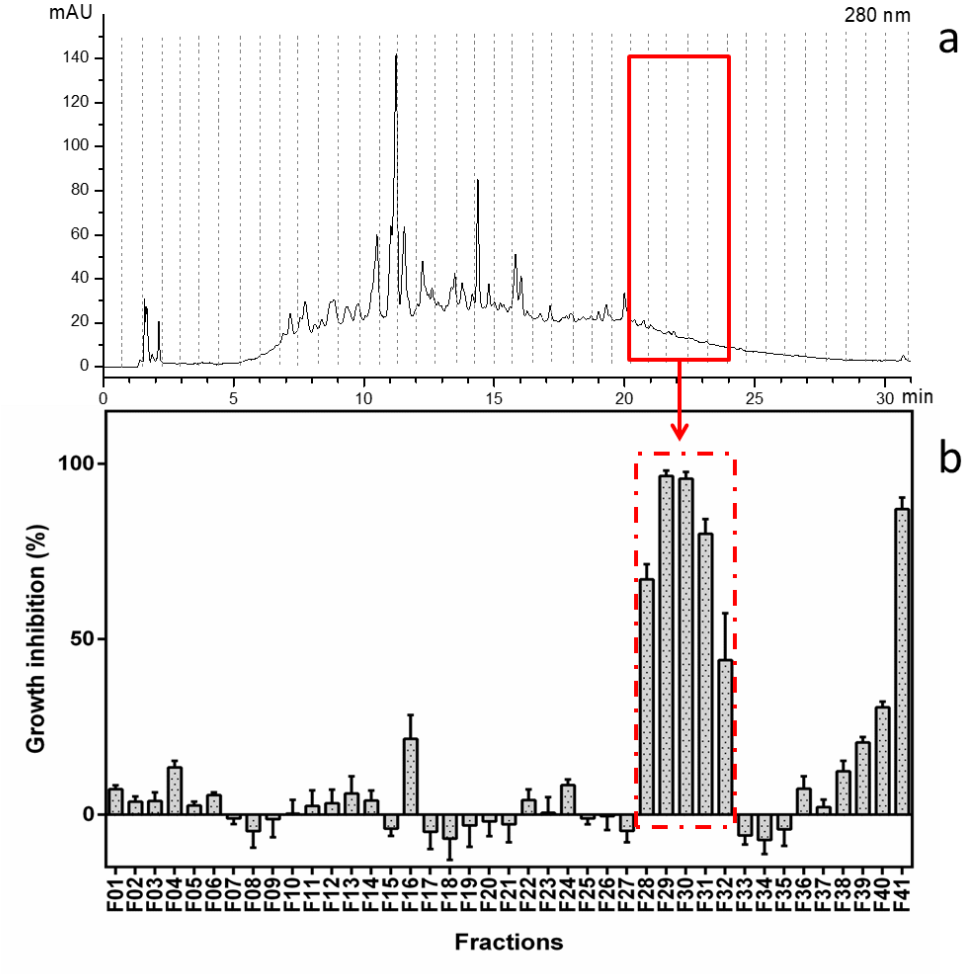

Supplement: Supplementary file 1 [file marinedrugs-17-00671-s001.pdf]
